# Full-Field Optical Coherence Tomography Using Galvo Filter-Based Wavelength Swept Laser

**DOI:** 10.3390/s16111933

**Published:** 2016-11-17

**Authors:** Muhammad Faizan Shirazi, Pilun Kim, Mansik Jeon, Jeehyun Kim

**Affiliations:** 1 School of Electronics Engineering, College of IT Engineering, Kyungpook National University, 80 Daehak-ro, Bukgu, 41566 Daegu, Korea; faizanshirazi110@gmail.com (M.F.S.); jeehk@knu.ac.kr (J.K.); 2 Oz-Tec Co. Ltd., Office 901, IT Convergence Industrial Building, 47 Gyeongdae-ro, 17-gil, Bukgu, 41566 Daegu, Korea; pukim@oz-tec.com

**Keywords:** OCT, wavelength swept laser, full-field OCT, large area scanning, galvo filter

## Abstract

We report a wavelength swept laser-based full-field optical coherence tomography for measuring the surfaces and thicknesses of refractive and reflective samples. The system consists of a galvo filter–based wavelength swept laser and a simple Michelson interferometer. Combinations of the reflective and refractive samples are used to demonstrate the performance of the system. By synchronizing the camera with the source, the cross-sectional information of the samples can be seen after each sweep of the swept source. This system can be effective for the thickness measurement of optical thin films as well as for the depth investigation of samples in industrial applications. A resolution target with a glass cover slip and a step height standard target are imaged, showing the cross-sectional and topographical information of the samples.

## 1. Introduction

With the rapid development of technology, the precision requirement in semiconductor chip technology, printed electronics, and various component industries has been increased owing to miniaturization. Additionally, the complexity of products has been increased using micro-processing technology. As a result, surface profile measurement has become an important issue in industrial inspection processes to reduce the defect rate and to fulfill the consumers’ demand. Many nondestructive three-dimensional (3D) surface profiling techniques have been developed to monitor product quality and to improve process efficiency [1,2,3,4]. Furthermore, different optical inspection methods have been proposed for 3D surface profile imaging with cost-effective, nondestructive, noncontact, high-speed, and high-accuracy solutions [5,6,7,8,9]. The amplitude variation is recorded by an optical interferometer with axial scanning methods utilizing the mechanical movement [6,7,8]. However, the susceptibility to hysteresis of the mechanical movement in imaging systems makes it unsuitable for profile measurement.

Optical coherence tomography (OCT) is a noninvasive and nondestructive technique for obtaining high-resolution tomographic images of microstructures [10]. OCT utilizes a Michelson interferometer with a low-coherence light source to obtain the cross-sectional depth information of an object. Using this method, three-dimensional images are obtained either in the time or frequency domain. Therefore, OCT is classified into two categories, the time domain and frequency domain [11]. The time domain (TD) OCT is relatively slow and has a low signal-to-noise ratio compared to the frequency domain (FD). The depth information obtained in the TD-OCT is because of the mechanical movement of the reference arm. In the frequency domain, the Fourier transform of the fringe patterns reveals the depth information of the sample in the FD-OCT [12,13]. In the frequency domain, the Fourier transform of the fringe patterns reveals the depth information of the sample. FD-OCT is further classified as spectral domain (SD) OCT and swept source (SS) OCT. In the SD-OCT, a broadband source is used for the illumination of the sample and reference, whereas the interference fringe is detected by a spectrometer. In the SS-OCT, a broadband swept source is employed to obtain the spectral fringes using a photodetector. Further, with the advancement in OCT, parallel acquisition of the interference fringe is possible by using an area camera in a full-field configuration; as a result, fast acquisition of the volume data is possible in the time domain as well as in the frequency domain [14,15]. The frequency domain full-field setup employs a wavelength swept laser for acquiring the interference signal from the sample and the reference arms without mechanical scanning, compared to the time domain. The frame rate in the frequency domain is a function of the sweep rate of the source and the camera acquisition speed.

OCT has extended its applications not only in biomedical imaging but also in agriculture, where it is used for the early detection of diseases in seeds and plants by investigating morphological abnormalities [16,17]. Numerous industrial applications of the OCT such as optical thin film, touch-screen panel assessment, liquid crystal display (LCD), and light-emitting diode (LED) inspections have been reported [18,19,20,21]. Similarly, various techniques have been employed in the OCT for increasing the image resolution, penetration depth, sensitivity, and field of view [22,23,24]. In the parallel area’s acquisition scheme, various samples have been investigated using a swept source with different filtering mechanisms and optical architecture. These samples include finger print detection, silicon integrated circuits, composite material reconstruction, micro-lens array measurement, in vivo human retina, ex vivo porcine eye, and tree shrew retina [25,26,27,28]. Similarly, wavelength scanning interferometry has been demonstrated for the profile measurement of transparent films and industrial objects [29,30,31,32]. In several applications, the thickness of a thin film including the measurements of both the top and bottom surfaces is critical. Similarly, in the semiconductor industry, a micrometer analysis includes the solder mask connection, through silicon via (TSV), and multilayer silicon wafer investigation.

In order to develop the wavelength swept laser, different filtering mechanisms have been used which includes the Fabry-Perot tunable filter, acousto-optic tunable filter, fiber Bragg grating, polygon mirror scanning filter, etc. Wavelength swept laser-based full-field OCT systems have several advantages compared to the other systems. One of the advantages is the parallel acquisition of signals using an area detector to get the area image of the sample. As a result, more power can be spread across the sample. The other advantage is that it does not need mechanical scanning in the sample arm to acquire a volume image. Because of the absence of x-y-z scanning, the full-field system is robust with high phase stability. The depth information is obtained by sweeping the wavelength in SS-OCT; the wavelength sweeping is done by the filtering mechanism incorporated in the source.

In this study, a simple, high-power galvo filter–based wavelength swept laser is integrated with a full-field OCT system for obtaining the tomographical as well as topographical features of the samples. The custom-built high-power wavelength swept laser is used to demonstrate the performance of the full-field OCT system for area scanning of samples. This study will contribute to the application of full-field OCT systems in the semiconductor industry for defect identification and investigation of samples.

## 2. Materials and Methods

### 2.1. Wavelength Swept Laser

**Figure 1:**
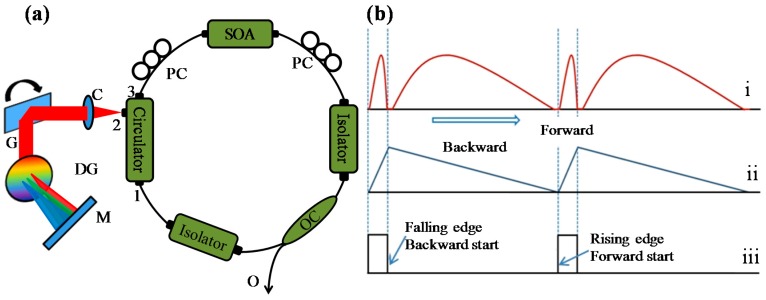
Schematic and timing diagram of the wavelength swept laser. (**a**) Schematic diagram of a galvo filter–based wavelength swept laser. C: collimator, DG: diffraction grating, G: galvo scanner, M: mirror, O: final output, OC: optical coupler, PC: polarization controller, SOA: semiconductor optical amplifier. (**b**) Timing of the main signals of the wavelength swept laser (i) scanning spectrum, (ii) waveform input to the galvo scanner driving board, and (iii) trigger signal at a 1 Hz frequency and a 10% duty cycle.

Figure 1a shows the schematic diagram of a galvo filter–based wavelength swept laser. The wavelength swept laser is composed of a fiber coupled semiconductor optical amplifier (SOA), two isolators, a 50:50 optical coupler, an optical circulator for the formation of a unidirectional ring cavity, two polarization controllers, and a galvo filter–based external cavity filter for selection of the lasing wavelength. The semiconductor optical amplifier (SOA-372, Superlum, Cork, Ireland) with maximum gain at a center wavelength of 848 nm is utilized as the broadband gain medium. All the components are connected in a ring cavity configuration through optical fibers. The SOAs have a high gain coefficient, robustness, and quick response characteristics in the gain medium for wavelength amplification.

The SOA has a broad amplified spontaneous emission (ASE) spectrum with 6 mW of optical power at a current of 145 mA. One side of the SOA is connected to the 50:50 optical fiber coupler through an isolator and the other side is connected to port-3 of the optical circulator. One of the output terminals of the coupler is connected to port-1 of the optical circulator through the other isolator, while the other output provides the wavelength swept laser spectrum. In this wavelength swept laser setup an external cavity is utilized as the band pass filter for selecting the wavelength. The wavelength selection filter is connected to port-2 of the optical circulator. The components used for wavelength selection in the external cavity configuration are a transmission type diffraction grating with 1800 lines per millimeter from Wasatch Photonics Inc. (Logan, UT, USA), a galvo scanner (GS001, Thorlabs, Newton, NJ, USA) and a mirror. The optical alignment of the filter is as follows: The output light from port-2 of the optical circulator is collimated and is incident on the center of the galvo scanning mirror after which it is reflected and passed through the diffraction grating, where it is dispersed into discrete wavelengths. The grating is aligned for providing the angular diffraction as a function of the input angle and wavelength with a maximum light intensity. As the grating’s grove direction is vertical, the incident light on the grating is diffracted horizontally.

The spectrally distributed light reflects back from the mirror placed at a centimeter distance from diffraction grating. This light follows the same return path and is coupled back to the optical circulator after passing through the grating, reflecting from the galvo mirror and is then incident on the collimator. Note that only the component of light that is normal to the mirror is coupled back to the fiber-based ring cavity. The galvo scanner is repeatedly scanned to produce the lasing output that results in an amplified optical signal at the output of the SOA. The isolators in the ring cavity ensure a unidirectional flow and protect the source by preventing reflections back from the application system to the cavity, to avoid catastrophic damages to the SOA. The sweeping output as a function of the galvo scanner frequency can be obtained from the output terminal of the optical coupler. The spectral line width of the feedback light is 0.05 nm with instantaneous coherence length of ~12 mm. The ring cavity length of the wavelength swept laser is approximately 2 m. This cavity length is acceptable for low sweep rate.

**Figure 2:**
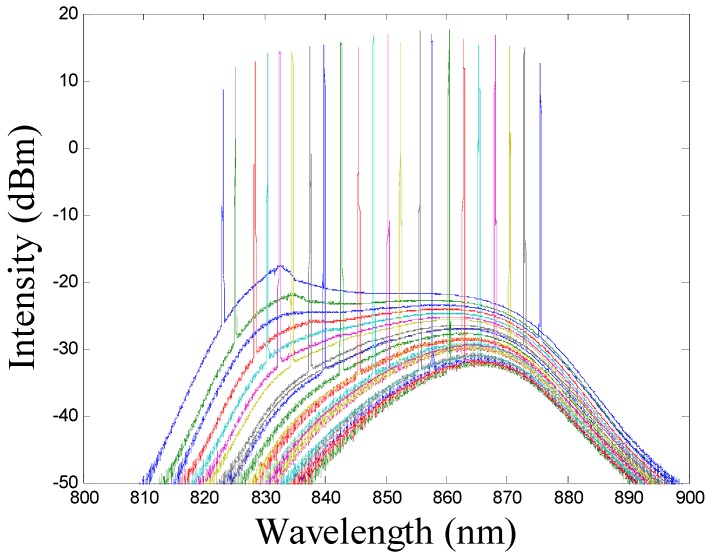
Swept source spectral bandwidth profile.

Figure 1b shows the input and output signal timing diagrams during the working of the wavelength swept laser. Three different signals are depicted: (i) the scanning spectrum of the wavelength swept laser, achieved when a (ii) triangular input signal is applied to the scan drive and simultaneously a (iii) trigger signal is generated. The arrow indicates the directions of the signals. The trigger signal has a 10% duty cycle that corresponds to the ramp-up duration of the triangular signal. The trigger signal can be synchronized to the application system for starting and stopping the data acquisition. In a single cycle of the input triangular signal to the galvo scanner drive, two sweeps are generated, as shown in (i). The forward and backward in (ii) indicate the voltage increment and decrement applied to the galvo scanner. In this wavelength swept laser the sweeping frequency, duty cycle, and the output power can be controlled by a customized electronic circuit with software control. This laser has a broad sweeping range with a 3 dB bandwidth of 48 nm as shown in Figure 2. Detailed information regarding this wavelength swept laser has been provided in a previous research [33]. The output profile of this source is assumed almost Gaussian. Therefore, the coherence length $\left(l_{c}\right)$ of the wavelength swept laser can be calculated using the following equation:

(1) $$lc=\frac{4\ln(2)}{\pi }\frac{\lambda_{0}^{2}}{\Delta \lambda }$$

From the above description of the source, we know that ∆λ = 48 nm and λ_0_ = 848 nm; therefore, substituting these values in Equation (1) will give us a coherence length of 13.184 µm. The axial resolution of the OCT system is half of *l_c_*; therefore, it is approximately 6.59 µm.

### 2.2. Full-Field OCT System

**Figure 3:**
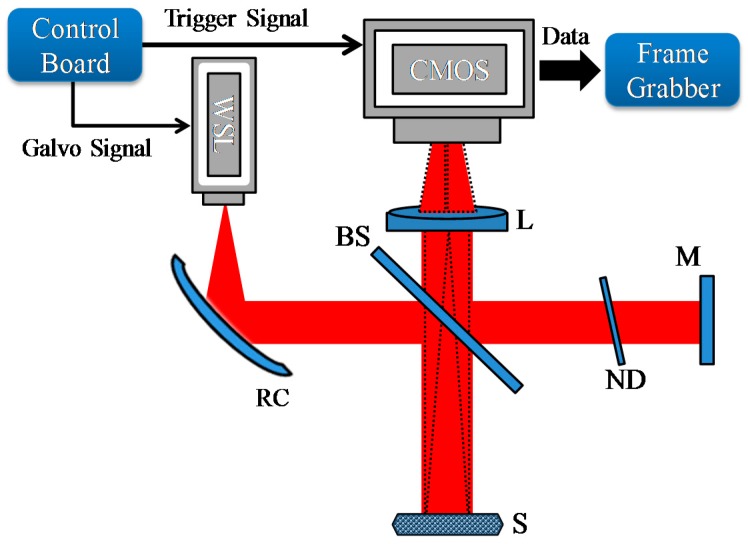
Schematic diagram of a full-field OCT system. BS: pellicle beam splitter, CMOS: complementary metal oxide semiconductor, L: lens, M: mirror, ND: neutral density filter, RC: reflective collimator, S: sample, WSL: wavelength swept laser.

The optical architecture of a wavelength swept laser-based full-field OCT system is shown in Figure 3. A Michelson interferometer setup is used to obtain an interference fringe signal by reflections from the sample and reference arms. The light from the wavelength swept laser is collimated using a metal-coated reflective parabolic mirror collimator (RC12APC-P01, Thorlabs, USA). The collimated light then passes through the beam splitter and it is divided into two paths. A portion of the light travel towards the sample and the remaining light is incident on the reference mirror. A variable neutral density filter is placed in the reference arm to decrease the intensity of the light in order to control the optical power in the reference arm. A continuously variable ND filter (NDC-50C-2M, Thorlabs, USA) is rotated to adjust the reflection from the reference arm in proportion to the back scattering from the sample. Data are acquired by a frame grabber through two camera link cables. A customized electronic control board is used to control the sweep frequency, SOA current, galvo signal, and the trigger signal.

An achromatic lens with a 30 mm focal length is utilized as the imaging lens for acquiring the raw fringe data from the sample and reference arms. The near infrared 8-bit area camera utilized here is the Basler ace (acA2000-340kmNIR, Basler, Ahrensburg, Germany) with a pixel resolution of 2048 × 1088 and maximum frame rate of 340 fps at full resolution. In order to avoid memory errors owing to large data and frame rate reduction problems, the region of interest is reduced to 500 × 500 pixels. The lateral resolution depends upon the numerical aperture of the lens and the pixel size of the camera. The axial resolution is a function of the spectral bandwidth of the source. The measured axial and lateral resolution of the system in current setup is ~10 µm and ~15 µm, respectively. The penetration depth depends on the wavelength and properties of sample. The sampling frequency of the camera also impacts the depth (axial) range. At sampling frequency of 340 fps, the wavelength sampling interval is ~0.15 nm which in turns gives the maximum imaging depth of ~2 mm. The system sensitivity drops off to 10 dB at 400 µm. The system has signal to noise ratio of 75 dB.

In the Fourier domain, the depth information can be obtained from the frequency components of the interference pattern after signal processing. If S(x,y;k) is the spectral density of the wavelength swept laser, $I_{R}$ and $I_{s}$ are the intensities of the reflected light from the reference and sample arms, respectively; hence, the total intensity detected at the camera is given as:

(2) $$\begin{array}{l} I\left(x,y;k\right)=S\left(x,y;k\right)I_{R}+S\left(x,y;k\right)\overset{\infty }{\underset{-\infty }{\iint }}\sqrt{I_{S}\left(z\right)I_{S}\left(z^{\prime }\right)}\exp\;\left(i\left\{k\left[\left(z-z^{\prime }\right)+\theta \left(z\right)-\theta \left(z^{\prime }\right)\right]\right\}\right)dzdz^{\prime }\\ +2S\left(x,y;k\right)\sqrt{I_{R}}\overset{\infty }{\underset{-\infty }{\int }}I_{S}\left(z\right)\cos\left[kz+\theta \left(z\right)\right]dz \end{array}$$

where *k* is the wave number, *z* is the depth, and θ(z) is the phase shift. The first two terms in Equation (2) are dc components; the first is generated by the intensity reflection from the reference mirror and the second is owing to the mutual interference between different layers of the sample. The third component is the result of the interference between the reference and sample signals. The cross-sectional depth information, *z*, of the sample can be retrieved from the third term after performing fast Fourier transform from the *k* to the *z* domain. Due to the non-linearity of the galvo filter, the interference signals are first resampled and linearized before Fourier transformation. The wavelength swept laser has phase stability with a standard deviation of ±0.01 radian.

### 2.3. Data Processing Steps

**Figure 4:**
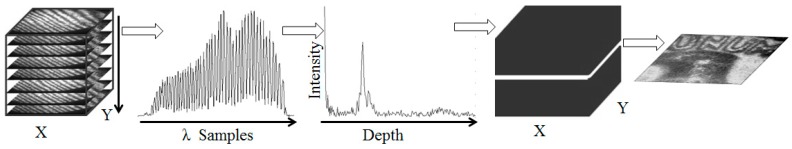
Signal processing steps (left to right) for acquiring the enface depth image. A stack of images is acquired by the camera along the wavelength axis, as indicated by the downward arrow. Next, a single interference signal is depicted with respect to the wavelength samples. A fast Fourier transform of this signal will provide the depth information. Hence, the respective enface depth image can be extracted from the 3D cube after processing all the acquired data.

For capturing images, a camera is synchronized with the wavelength swept laser using a trigger signal. The frame grabber utilized in this system is the PCIe-1433 (National Instruments, Austin, TX, USA) and a camera link full configuration is used to run the camera at a maximum frame rate. Figure 4 shows the signal processing mechanism employed for obtaining the OCT images in a wavelength swept laser based full-field OCT system. The mechanism is initiated by the acquisition of raw area images from the camera in the wavelength scanning direction of the wavelength swept laser. The camera starts the acquisition with the falling edge of the trigger and continues up to the rising edge of the trigger. All the acquired frames contain the interference fringe information along with the number of frames. Fast Fourier transform (FFT) of this acquired signal will provide the depth information. The data is resampled and linearized before FFT to avoid wave number non-linearization due to the non-linearity of galvo filter. In the case of the mirror, a single peak represents the mirror position as a function of the path difference between the two arms. In the case of complex samples, multiple peaks are obtained in the Fourier spectrum. By applying FFT for each interference signal, all the frames are processed and the resulting enface images at different depths can be seen (Figure 4). Similarly, by selective filtering of the peaks, the amplitude and phase information of a particular surface can be extracted with a micrometer to nanometer resolution. A LabVIEW program is coded to acquire the data and view the real-time cross-sectional image with the depth information. For enface OCT images, a program is coded to post-process the saved data. As mentioned earlier that the area image has dimension of 500 × 500 pixels with 340 frames per second. Therefore, the system has B-scan frame rate of 500 with each frame contains 500 A-scans and each A-scan contains 340 sample points.

## 3. Results and Discussion

**Figure 5:**
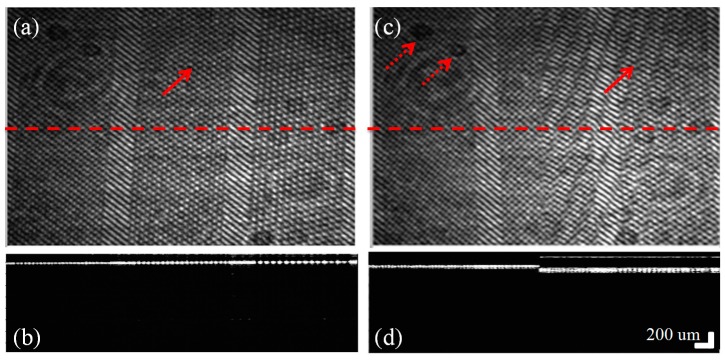
Tomographic images of the full-field OCT system. (**a**,**c**) Raw enface images of the Edmund optics target and the Edmund optics target with a 170-μm-thick cover slip, respectively. (**b**,**d**) Cross-sectional images, clearly distinguishing the line separations along the dashed lines in (**a**,**c**), respectively. The solid arrow in (**a**) shows the interference fringe because of the reference mirror and the resolution target, while in (**c**) the arrow indicates the fringe pattern owing to the cover slip, reference mirror, and resolution target. The dotted arrow shows the background noise on the sample.

Figure 5 shows the cross-sectional images using the proposed wavelength swept laser-based full-field OCT system. The wavelength swept laser is operated at 1 Hz with two sweep spectrums corresponding to a 10% forward sweep and a 90% backward sweep, as shown in (i) of Figure 1b. The camera starts the acquisition at the falling edge of the trigger signal connected to the frame grabber. The data acquired in the 90% backward sweep is processed in the 10% forward sweep duration and simultaneously displayed. Figure 5a,c show the raw area images of an Edmund optics target and an Edmund optics target with a 170-μm-thick cover slip, respectively. Similarly, Figure 5b,d are the cross-sectional images along the red-colored dashed line of the respective targets. Different fringe patterns appear in regions with and without the cover slip in Figure 5c. The fringe pattern is obtained as a result of the interference between the sample and reference arms, provided that the path difference is within the coherence length of the source. In Figure 5c, the fringe pattern in the right half is due to the interference between the reference arm and light reflected from the top of the cover slip and the Edmond optics target. The Edmund optics target has various lines per millimeter (l pmm), starting from five and reaching up to 200. In this experiment, the portions containing 25, 20, and 15 lines per millimeter are imaged with a field of view of 3 mm × 3 mm, corresponding to 500 × 500 camera pixels. In Figure 5b, the line separation can be seen clearly, corresponding to a lateral distance of 20 μm according to the target. The refractive index of the cover slip is 1.52 μm. The separation between the cover slip and the target can be clearly seen in Figure 5d, corresponding to an optical thickness of 112 μm. Owing to the change in the refractive index, there is a shift in the position of the surface of the resolution target, as can be seen at the intersection. The solid arrows indicate the fringe pattern, while the dashed arrows point to the background noise. Using this experiment, the tomographical thickness variation in the samples can be observed, and with calibration, an accurate measurement can be computed.

**Figure 6:**
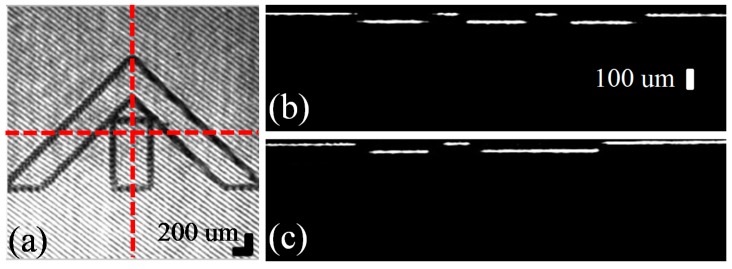
Tomographic images of the full-field OCT system; (**a**) Raw enface image of the VLSI 50 µm step height standard target; (**b**,**c**) Cross-sectional profiles along the horizontal and vertical red-colored dashed lines in (**a**), respectively.

The next experiment is performed for visualizing the topological information of a very large scale integration (VLSI) height standard target with a step height of 50 μm. Figure 6a shows a 3 mm × 3 mm portion of the standard mark on the VLSI target. The height variations are shown in Figure 6b,c along the vertical and horizontal cross-sections, respectively, of the red-colored dashed line in Figure 6a. These tomographical features can be seen in entirety in real time using the proposed system. For visualizing and detecting defects in industrial samples with refractive and reflective characteristics, the proposed system can be effective and can generate fault detection results with microscopic resolutions. Similarly, tomographical and topographical variation in samples can be precisely monitored using this system.

**Figure 7:**
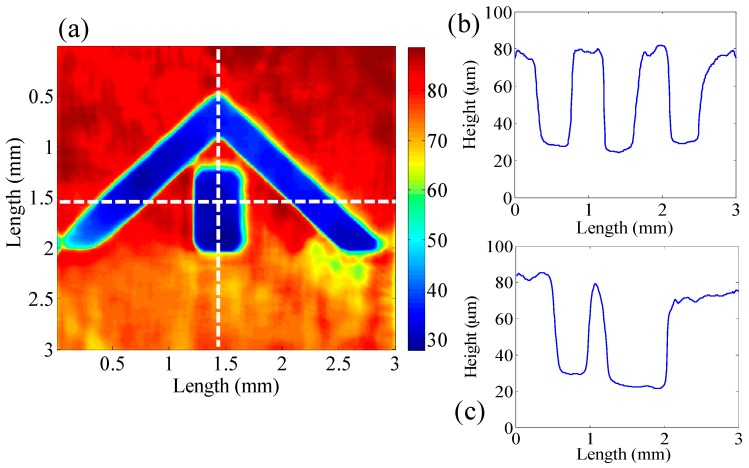
Tomographic image of the full-field OCT system; (**a**) Enface image of the VLSI 50 µm step height standard target; (**b**,**c**) Cross-sectional profiles along the horizontal and vertical white-colored dashed lines in (**a**), respectively.

Figure 7 shows the enface image of a portion of the VLSI target. The color-coded image in Figure 7a shows the topographical variations with an almost 50 μm step height with a 3 mm × 3 mm field of view. Figure 7b,c show the height variation along the horizontal and vertical white dashed line in Figure 7a, respectively. By utilizing this information, the topographical variations in industrial samples can be monitored with high precision. The system has height variation within the standard deviation of ±1 µm.

In future works, the performance of this system can be improved by utilizing the 4*f* configuration in the detection part of the camera. By increasing the sweep duration of the wavelength swept laser, more images can be acquired to improve the imaging depth of the system. The further improvement of the system can be done by increasing the sweep rate of the source with a high speed camera. The spatial interference of light reflected from the sample affects the transverse resolution of the system. Therefore, the transverse resolution of the system can be improved by destroying the spatial coherence of the illumination beam by utilizing the diffuser or mode mixer.

## 4. Conclusions

We have demonstrated the tomographical and topographical features of samples using the wavelength swept laser-based full-field optical coherence tomography system. The Edmund optics target with a transparent cover slip indicates the lateral resolution and thickness of the cover slip. The optical arrangement is realized by using a high-power wavelength swept laser. The simple proposed optical system can play an important role in the inspection of industrial samples. The system has a comparatively large field of view, is easy to align with non-mechanical scanning components, and has a compact optical setup. In future works, this system will be used for measuring the varying thicknesses of optical thin films including both the top and bottom surfaces. Solder mask connection tests, the connectivity of through silicon vias, and multilayer silicon wafer investigations in the semiconductor industry are the areas in which the system performance can be further evaluated.
